# Emergence of Beta Oscillations of a Resonance Model for Parkinson's Disease

**DOI:** 10.1155/2020/8824760

**Published:** 2020-11-30

**Authors:** Yaqian Chen, Junsong Wang, Yanmei Kang, Muhammad Bilal Ghori

**Affiliations:** ^1^School of Mathematics and Statistics, Xi'an Jiaotong University, Xi'an, 710049 Shaanxi Province, China; ^2^School of Biomedical Engineering, Tianjin Medical University, Tianjin 300070, China

## Abstract

In Parkinson's disease, the excess of beta oscillations in cortical-basal ganglia (BG) circuits has been correlated with normal movement suppression. In this paper, a physiologically based resonance model, generalizing an earlier model of the STN-GPe circuit, is employed to analyze critical dynamics of the occurrence of beta oscillations, which correspond to Hopf bifurcation. With the experimentally measured parameters, conditions for the occurrence of Hopf bifurcation with time delay are deduced by means of linear stability analysis, center manifold theorem, and normal form analysis. It is found that beta oscillations can be induced by increasing synaptic transmission delay. Furthermore, it is revealed that the oscillations originate from interaction among different synaptic connections. Our analytical results are consistent with the previous experimental and simulating findings, thus may provide a more systematic insight into the mechanisms underlying the transient beta bursts.

## 1. Introduction

Parkinson's disease ranks as the second most common neurodegenerative disorder after Alzheimer's disease [1–3]. According to data from the Parkinson's Foundation [4], more than 10 million people worldwide are suffering from the typical motor symptoms including bradykinesia, muscular rigidity, rest tremor, and postural and gait impairment, as well as nonmotor impairment such as mood and sleep disorders, cognitive decline, urinary symptoms, and incontinence [5, 6]. With an incidence ranging from 10–18 per 100000 person years [5], the health of increasingly individuals is debilitated.

A commonly acknowledged opinion is that these symptoms are associated with the degeneration of dopaminergic neurons in the substantia nigra par compacta, which releases the neurotransmitter dopamine to the basal ganglia [7]. Particularly, excessive beta oscillations (13-30 Hz) have been observed in the local field potential recording from the basal ganglia of Parkinson patients with symptom severity [6–9]. The magnitude of beta oscillations is even related to the severity and degree of bradykinetic or akinetic motor symptoms and rigidity [10, 11]. Some motor symptoms can be ameliorated by suppressing these oscillations by deep brain stimulation (DBS) or dopaminergic medications [12–14]. The recent clinical findings are persuading more attention to deeply explore the mechanisms of beta oscillations associated with Parkinson's disease.

Several computational models have been proposed to elucidate the generation of pathologically exaggerated beta oscillations in Parkinson's disease [15–21]. With focus on the subthalamic nucleus-globus pallidus circuit, Holgado et al. developed a mean-field model and suggested that beta oscillations observed in the basal ganglia arise due to interactions of two nuclei: the subthalamic nucleus (STN) and the globus pallidus pars externa (GPe) [15]. They also found that the Hopf bifurcation can occur when increasing the synaptic weights from healthy to Parkinsonian regimes. Hu et al. [17] used an improved network model consisting of two STN populations and one GP population. Some recent experiments evidenced that in addition to the STN-GP loop, the motor cortex also plays a critical role in the generation of pathological beta oscillations in Parkinson's disease [10, 13, 22, 23]. Pavlides et al. [24] thus proposed a cortex-subthalamic nucleus-globus pallidus model, with the excitatory and inhibitory neuronal populations of the cortex taken into account, and successfully reproduced the beta oscillations in the experimental observation by Tachibana et al. [25]. Since it was proposed under the hypothesis that beta oscillations are generated in the motor cortex, and the basal ganglia resonate to the cortical input, the cortex-subthalamic nucleus-globus pallidus model is also called as resonance model. For brevity, we refer to it as the resonance model from now on.

Note that the resonance model is mainly explored by data fitting and parameter identification [24], thus the fundamental dynamical mechanism by which the beta oscillation is generated has not been disclosed. According to the research with the STN-GP circuit [16], it was found that Hopf bifurcation can induce the pathologically exaggerated beta oscillations. Thus, we wonder whether this is still the dynamical mechanism for the resonance model. We also note that synaptic transmission, the biological process by which a neuron communicates with a target cell across a synapse, always plays a critical part in the neuronal activity [3, 26]. For instance, blocking excitatory synaptic transmission could decrease neural firing and reveal spontaneous firing [26]. In particular, experimental evidence suggests that the loss of dopamine in Parkinson's disease could influence dendritic excitability [27]. Recent model investigations [16, 17, 26] further highlighted that higher synaptic transmission delays and strong synaptic connections between STN and GP populations are beneficial for promoting beta-frequency activity. Therefore, in the present study, we aim to validate the Hopf bifurcation mechanism of the pathological beta oscillation in the resonance model based on the normal form theory, using synaptic transmission delay and synaptic connection weight as bifurcation parameters.

## 2. Materials and Methods

### 2.1. The Model and Existence of Hopf Bifurcation

**Figure 1:**
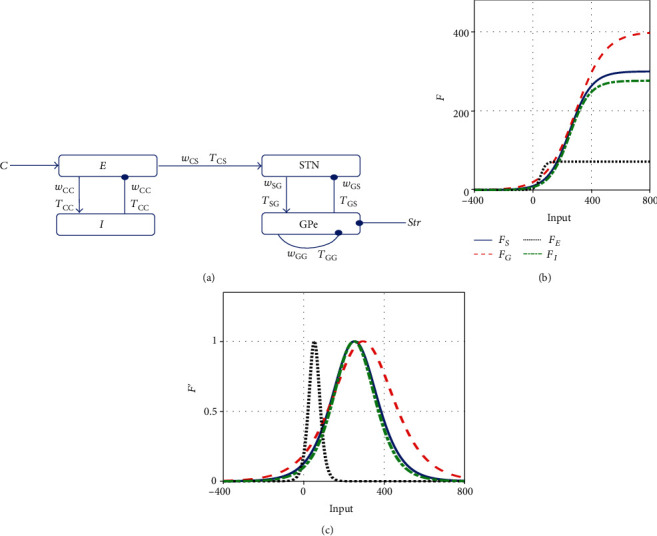
(a) Connectivity in the cortical-basal-ganglia-thalamic circuit in the resonance model. This model includes sigmoid functions for STN, GPe, excitatory, and inhibitory populations, which describe the input-output relationship of neurons in the populations as shown in Figure 1(b). Here, *w*_*ij*_ and *T*_*ij*_ denote the connection strength and synaptic delay between neural populations *i* and *j*, respectively. The arrows represent the excitatory input, and the solid points represent the inhibitory input. (b) Output from the activation function *F*_*X*_(in)(*X* = *S*, *G*, *E*, *I*), given by Eq. (2). (c) Derivatives of the activation function from Figure 1(b).

We begin with a review of the resonance model proposed by Alex Pavlides et al. [24], which investigated the cortico-basal–gangalia-thalamic circuit as depicted in Figure 1(a). The model includes two circuits, one composed of interconnected neural populations in STN and GPe, and another composed of excitatory and inhibitory neurons in the cortex. Moreover, the excitatory cortical neurons project excitatory glutamatergic axons to STN. The model is described by a continuum mean-field approach, given by

(1) $$\begin{array}{c} \begin{array}{c} \tau_{S}S^{\prime }=F_{S}\left(w_{CS}E\left(t-T_{CS}\right)-w_{GS}G\left(t-T_{GS}\right)\right)-S\left(t\right),\\ \tau_{G}G^{\prime }=F_{G}\left(w_{SG}S\left(t-T_{SG}\right)-w_{GG}G\left(t-T_{GG}\right)-Str\right)-G\left(t\right),\\ \tau_{E}E^{\prime }=F_{E}\left(-w_{CC}I\left(t-T_{CC}\right)+C\right)-E\left(t\right),\\ \tau_{I}I^{\prime }=F_{I}\left(w_{CC}E\left(t-T_{CC}\right)\right)-I\left(t\right), \end{array} \end{array}$$

where *S*(*t*), *G*(*t*), *E*(*t*), and *I*(*t*) are the firing rates of STN, GPe, excitatory, and inhibitory populations, respectively. Str denotes the constant inhibitory input from striatum to GPe, and *C* denotes the constant component of extrinsic and intrinsic excitatory input to cortical excitatory neurons. The parameters *T*_*ij*_ and *w*_*ij*_ represent transmission delay and connection weight, respectively. Here, the subscript *i* indicates the population from which the signal originates, and the subscript *j* indicates where the signal is received. *τ*_*x*_ denotes the membrane time constants for population *x*, describing how rapidly the population reacts to its inputs. Notice that the “resonance” is mainly reflected in the hypothesis that the oscillations in basal ganglia resonate to the excitatory cortical input.

The terms *F*_*X*_(*X* = *S*, *G*, *E*, *I*) are the sigmoid activation functions expressing the relationship between firing rate and synaptic input, shown as follows

(2) $$\begin{array}{c} F_{X}\left(\text{in}\right)=\frac{M_{X}}{1+\left(\left(M_{X}-B_{X}\right)/B_{X}\right)\exp\left(-4\text{in}/M_{X}\right)},\left(X=S,G,E,I\right). \end{array}$$

Here, the constant *M*_*X*_ is the maximum firing rate of population *X*, and *B*_*X*_ is the firing rate in the absence of the synaptic input. The curves of these activation functions and their derivatives are shown in Figures 1(b) and 1(c), respectively.

The values of all parameters in this paper, except for the transmission delay and connection weights, are summarized in Table 1. It is noteworthy that these values could be estimated directly on the basis of experimental data, more details could be found in Ref. [24].

It is well known that one of the classical mechanism for occurring oscillations is the Hopf bifurcation, in which the attractive limit cycle and asymptotically stable equilibrium point could transform as parameters change. And these two states correspond to the Parkinsonian and healthy state in the STN-GPe model, respectively. Thus, we will investigate the conditions for Hopf bifurcation of the resonance model here. Since the model is difficult to analyze mathematically, we make two simplifications: (i) the membrane time constants *τ*_*X*_(*X* = *S*, *G*, *E*, *I*) were taken to have an average value of *τ* = 10ms; (ii) all transmission delays were taken to be equal, denoted by the single variable *T*. Then, we could get the following system:

(3) $$\begin{array}{c} \begin{array}{c} \tau S^{\prime }=F_{S}\left(w_{CS}E\left(t-T\right)-w_{GS}G\left(t-T\right)\right)-S\left(t\right),\\ \tau G^{\prime }=F_{G}\left(w_{SG}S\left(t-T\right)-w_{GG}G\left(t-T\right)-Str\right)-G\left(t\right),\\ \tau E^{\prime }=F_{E}\left(-w_{CC}I\left(t-T\right)+C\right)-E\left(t\right),\\ \tau I^{\prime }=F_{I}\left(w_{CC}E\left(t-T\right)\right)-I\left(t\right). \end{array} \end{array}$$

denoting the equilibrium point of the system as *u*_0_ = (*S*^∗^, *G*^∗^, *E*^∗^, *I*^∗^)^*T*^, and the model (3) around the equilibrium point can be formally rewritten into

(4) $$\begin{array}{c} \frac{du}{dt}=B_{1}u\left(t\right)+B_{2}u\left(t-T\right)+f\left(u\left(t-T\right)\right), \end{array}$$

with *u*(*t*) = (*S*(*t*) − *S*^∗^, *G*(*t*) − *G*^∗^, *E*(*t*) − *E*^∗^, *I*(*t*) − *I*^∗^)^*T*^. Here, *f* = (*f*_*S*_, *f*_*G*_, *f*_*E*_, *f*_*I*_)^*T*^ is deduced from the higher-order terms of the vector field and *B*_1_ = −(1/*τ*)*I*_4×4_

(5) $$\begin{array}{c} B_{2}=\left[\begin{array}{cccc} 0 & a_{12} & a_{13} & 0\\ a_{21} & a_{22} & 0 & 0\\ 0 & 0 & 0 & a_{34}\\ 0 & 0 & a_{43} & 0 \end{array}\right], \end{array}$$

with

(6) $$\begin{array}{c} a_{12}=\frac{4S^{*2}w_{GS}}{\tau M_{S}^{2}}-\frac{4S^{*}w_{GS}}{\tau M_{S}},a_{13}=\frac{-4S^{*2}w_{CS}}{\tau M_{S}^{2}}+\frac{4S^{*}w_{CS}}{\tau M_{S}},\\ a_{21}=\frac{-4G^{*2}w_{SG}}{\tau M_{G}^{2}}+\frac{4G^{*}w_{SG}}{\tau M_{G}},a_{22}=\frac{4G^{*2}w_{GG}}{\tau M_{G}^{2}}-\frac{4G^{*}w_{GG}}{\tau M_{G}},\\ a_{34}=\frac{4E^{*2}w_{CC}}{\tau M_{E}^{2}}-\frac{4E^{*}w_{CC}}{\tau M_{E}},a_{43}=\frac{-4I^{*2}w_{CC}}{\tau M_{I}^{2}}+\frac{4I^{*}w_{CC}}{\tau M_{I}}. \end{array}$$

For the linearized system *du*/*dt* = *B*_1_*u*(*t*) + *B*_2_*u*(*t* − *T*), the characteristic equation is given by

(7) $$\begin{array}{c} \begin{array}{c} \Delta \left(\lambda \right)=\left|\begin{array}{cccc} \lambda +1/\tau & -a_{12}\exp\left(-\lambda T\right) & -a_{13}\exp\left(-\lambda T\right) & 0\\ -a_{21}\exp\left(-\lambda T\right) & \lambda +1/\tau -a_{22}\exp\left(-\lambda T\right) & 0 & 0\\ 0 & 0 & \lambda +1/\tau & -a_{34}\exp\left(-\lambda T\right)\\ 0 & 0 & -a_{43}\exp\left(-\lambda T\right) & \lambda +1/\tau \end{array}\right|\\ =\Delta_{1}\left(\lambda \right)\Delta_{2}\left(\lambda \right)\\ =0, \end{array} \end{array}$$

where

(8) $$\begin{array}{c} \Delta_{1}\left(\lambda \right)={\left(\lambda +1/\tau \right)}^{2}-\left(\lambda +1/\tau \right)a_{22}\exp\left(-\lambda T\right)-a_{12}a_{21}\exp\left(-2\lambda T\right),\\ \Delta_{2}\left(\lambda \right)={\left(\lambda +\frac{1}{\tau }\right)}^{2}-a_{34}a_{43}\exp\left(-2\lambda T\right) \end{array}$$

represent filtering that occurs when a signal gets through the STN-GPe loop and the cortical excitatory-inhibitory loop, respectively. Thus, the linear instability for Hopf bifurcation can be induced by resonance in filter of the STN-GPe loop or cortical excitatory-inhibitory loop.

As well known, if Eq. (7) has a pair of purely imaginary roots ±*iω*(*ω* > 0), the stability should be changed, and a Hopf bifurcation may occurs. So, we consider Δ_1_(*iω*) = 0 and Δ_2_(*iω*) = 0 next.

- Suppose that *λ* = *iω*(*ω* > 0) is a root of Δ_1_(*λ*), i.e.,

(9) $$\begin{array}{c} \Delta_{1}\left(\lambda \right)\exp\left(\lambda T\right)=-i\omega a_{22}-\frac{a_{22}}{\tau }+\left(-\omega^{2}+\frac{1}{\tau^{2}}+i\frac{2\omega }{\tau }\right)\left(\cos\omega T+i\sin\omega T\right)-a_{12}a_{21}\left(\cos\omega T-i\sin\omega T\right)=0. \end{array}$$

Separating real parts and imaginary parts of Eq. (9), one can obtain

(10) $$\begin{array}{c} \left(-\omega^{2}+\frac{1}{\tau^{2}}\right)\sin\omega T+\frac{2\omega }{\tau }\cos\omega T=\omega a_{22}-a_{12}a_{21}\sin\omega T,\\ \left(-\omega^{2}+\frac{1}{\tau^{2}}\right)\cos\omega T-\frac{2\omega }{\tau }\sin\omega T=\frac{a_{22}}{\tau }+a_{12}a_{21}\cos\omega T. \end{array}$$

Then, one can further obtain

(11) $$\begin{array}{c} \sin\omega T=\frac{-a_{22}\tau^{4}\omega^{3}-\left(\tau^{2}a_{22}+\tau^{4}a_{12}a_{21}a_{22}\right)\omega }{\tau^{4}\omega^{4}+2\tau^{2}\omega^{2}+1-\tau^{4}a_{12}^{2}a_{21}^{2}}, \end{array}$$

(12) $$\begin{array}{c} \cos\omega T=\frac{a_{22}\tau^{3}\omega^{2}+\tau a_{22}+\tau^{3}a_{12}a_{21}a_{22}}{\tau^{4}\omega^{4}+2\tau^{2}\omega^{2}+1-\tau^{4}a_{12}^{2}a_{21}^{2}}. \end{array}$$

According to Eqs. (11) and (12) and sin^2^(*ωT*) + cos^2^(*ωT*) = 1, we have

(13) $$\begin{array}{c} \omega^{8}+k_{1}\omega^{6}+k_{2}\omega^{4}+k_{3}\omega^{2}+k_{4}=0, \end{array}$$

where

(14) $$\begin{array}{c} k_{1}=4/\tau^{2}-a_{22}^{2},\\ k_{2}=\frac{6}{\tau^{4}}-\frac{3a_{22}^{2}}{\tau^{2}}-2a_{12}a_{21}\left(a_{12}a_{21}+a_{22}^{2}\right),\\ k_{3}=\frac{4}{\tau^{6}}-\frac{3a_{22}^{2}}{\tau^{4}}-\frac{4a_{12}a_{21}\left(a_{12}a_{21}+a_{22}^{2}\right)}{\tau^{2}}-{\left(a_{12}a_{21}a_{22}\right)}^{2},\\ k_{4}=\frac{1}{\tau^{8}}-\frac{a_{22}^{2}}{\tau^{6}}-\frac{2a_{12}a_{21}\left(a_{12}a_{21}+a_{22}^{2}\right)}{\tau^{4}}-\frac{{\left(a_{12}a_{21}a_{22}\right)}^{2}}{\tau^{2}}+{\left(a_{12}a_{21}\right)}^{4}. \end{array}$$

Let *z* = *ω*^2^, then Eq. (13) becomes

(15) $$\begin{array}{c} z^{4}+k_{1}z^{3}+k_{2}z^{2}+k_{3}z+k_{4}=0. \end{array}$$

Here, we give the following assumption (H1). Eq. (15) has at least one positive root. Without loss of generality, we assume that it has four positive roots, denoting by *z*_*k*_, (*k* = 1, 2, ⋯, 4). Then, Eq. (13) has four positive roots as well, namely, $\omega_{k}=\sqrt{z_{k}},\left(k=1,2,\cdots ,4\right)$. According to Eq. (12), we can get the corresponding critical value of time delay

(16) $$\begin{array}{c} T_{k}^{j}=\frac{1}{\omega_{k}}\arccos\frac{a_{22}\tau^{3}\omega_{k}^{2}+\tau a_{22}+\tau^{3}a_{12}a_{21}a_{22}}{\tau^{4}\omega_{k}^{4}+2\tau^{2}\omega_{k}^{2}+1-\tau^{4}a_{12}^{2}a_{21}^{2}}+\frac{2j\pi }{\omega_{k}},k=1,2,3,4;\;j=0,1,2,\cdots \end{array}$$

Then ±*iω*_*k*_ is a pair of purely imaginary roots of Eq. (7) with *T*_*k*_^*j*^.

(ii) Suppose that *λ* = *iω*(*ω* > 0) is a root of Δ_2_(*λ*), i.e.,

(17) $$\begin{array}{c} \omega^{4}+\frac{2\omega^{2}}{\tau^{2}}+\frac{1}{\tau^{4}}-{\left(a_{34}a_{43}\right)}^{2}=0. \end{array}$$

So, we know that $\omega =\sqrt{-a_{34}a_{43}-1/\tau^{2}}$ and the corresponding time delay are

(18) $$\begin{array}{c} T_{5}^{j}=\frac{-1}{2\omega }\arcsin\frac{2\omega }{\tau a_{34}a_{43}}+\frac{j\pi }{\omega },j=0,1,2,\cdots \end{array}$$

Finally, let the critical time delay as $T_{0}=\underset{k=\left\{1,2,3,4,5\right\}}{\min}\left\{T_{k}^{j}\right\}$ and the corresponding purely imaginary roots as ±*iω*_0_.

Next, we need to verify the transversality condition. Differentiating the two sides of Eq. (7) on time delay, we have

(19) $$\begin{array}{c} {\left[\frac{d\lambda }{dT}\right]}^{-1}=-\frac{d_{1}\Delta_{1}\left(\lambda \right)+d_{2}\Delta_{2}\left(\lambda \right)}{d_{3}\Delta_{1}\left(\lambda \right)+d_{4}\Delta_{2}\left(\lambda \right)}, \end{array}$$

where

(20) $$\begin{array}{c} \begin{array}{c} d_{1}=2\left(\lambda +1/\tau +{Ta}_{34}a_{43}\exp\left(-2\lambda T\right)\right),\\ d_{2}=2\left(\lambda +1/\tau \right)+a_{22}\exp\left(-\lambda T\right)\left(T\lambda +T/\tau -1\right)+2{Ta}_{12}a_{21}\exp\left(-2\lambda T\right),\\ d_{3}=2\lambda a_{34}a_{43}\exp\left(-2\lambda T\right),\\ d_{4}=\lambda \left(\lambda +1/\tau \right)a_{22}\exp\left(-\lambda T\right)+2\lambda a_{12}a_{21}\exp\left(-2\lambda T\right). \end{array} \end{array}$$

Thus,

(21) RedλdTλ=iω0−1=−PRQR+PIQIQR2+QI2,

with

(22) $$\begin{array}{c} P_{R}=-d_{1\text{I}}\Delta_{1\text{I}}+d_{1\text{R}}\Delta_{1\text{R}}-d_{2\text{I}}\Delta_{2\text{I}}+d_{2\text{R}}\Delta_{2\text{R}},\\ P_{I}=d_{1\text{I}}\Delta_{1\text{R}}+d_{1\text{R}}\Delta_{1\text{I}}+d_{2\text{I}}\Delta_{2\text{R}}+d_{2\text{R}}\Delta_{2\text{I}},\\ Q_{R}=-d_{3\text{I}}\Delta_{1\text{I}}+d_{3\text{R}}\Delta_{1\text{R}}-d_{4\text{I}}\Delta_{2\text{I}}+d_{4\text{R}}\Delta_{2\text{R}},\\ Q_{I}=d_{3\text{I}}\Delta_{1\text{R}}+d_{3\text{R}}\Delta_{1\text{I}}+d_{4\text{I}}\Delta_{2\text{R}}+d_{4\text{R}}\Delta_{2\text{I}},\\ d_{1\text{I}}=2\omega_{0}-2{Ta}_{34}a_{43}\sin\left(2\omega_{0}T\right),d_{1\text{R}}=2/\tau +2{Ta}_{34}a_{43}\cos\left(2\omega_{0}T\right),\\ d_{2\text{I}}=2\omega_{0}+{Ta}_{22}\omega_{0}\cos\left(\omega_{0}T\right)-a_{22}\left(T/\tau -1\right)\sin\left(\omega_{0}T\right)-2{Ta}_{12}a_{21}\sin\left(2\omega_{0}T\right),\\ d_{2\text{R}}=2/\tau +{Ta}_{22}\omega_{0}\sin\left(\omega_{0}T\right)+a_{22}\left(T/\tau -1\right)\cos\left(\omega_{0}T\right)+2{Ta}_{12}a_{21}\cos\left(2\omega_{0}T\right),\\ d_{3\text{I}}=2\omega_{0}a_{34}a_{43}\cos\left(2\omega_{0}T\right),d_{3\text{R}}=2\omega_{0}a_{34}a_{43}\sin\left(2\omega_{0}T\right),\\ d_{4\text{I}}=\frac{\omega_{0}a_{22}}{\tau }\cos\left(\omega_{0}T\right)+\omega_{0}^{2}a_{22}\sin\left(\omega_{0}T\right)+2\omega_{0}a_{12}a_{21}\cos\left(2\omega_{0}T\right),\\ d_{4\text{R}}=-\omega_{0}^{2}a_{22}\cos\left(\omega_{0}T\right)+\frac{\omega_{0}a_{22}}{\tau }\sin\left(\omega_{0}T\right)+2\omega_{0}a_{12}a_{21}\sin\left(2\omega_{0}T\right),\\ \Delta_{1\text{I}}=\frac{2\omega_{0}}{\tau }-\omega_{0}a_{22}\cos\left(\omega_{0}T\right)+\frac{a_{22}}{\tau }\sin\left(\omega_{0}T\right)+a_{12}a_{21}\sin\left(2\omega_{0}T\right),\\ \Delta_{1\text{R}}=-\omega_{0}^{2}+\frac{1}{\tau^{2}}-\omega_{0}a_{22}\sin\left(\omega_{0}T\right)-\frac{a_{22}}{\tau }\cos\left(\omega_{0}T\right)-a_{12}a_{21}\cos\left(2\omega_{0}T\right),\\ \Delta_{2\text{I}}=\frac{2\omega_{0}}{\tau }+a_{34}a_{43}\sin\left(2\omega_{0}T\right),\Delta_{2\text{R}}=-\omega_{0}^{2}+\frac{1}{\tau^{2}}-a_{34}a_{43}\cos\left(2\omega_{0}T\right). \end{array}$$

Therefore, if (H2) *P*_*R*_*Q*_*R*_ + *P*_*I*_*Q*_*I*_ ≠ 0 holds, Re{(*dλ*/*dT*)_*λ*=*iω*_0__^−1^} ≠ 0, the transversality condition for Hopf bifurcation is satisfied.

On the other hand, we need to prove that the remaining roots of Eq. (7) have strictly negative real parts. The following Lemma is used.

**Lemma 1 .**

[28, 29]. Consider the exponential polynomial

(23) $$\begin{array}{c} \begin{array}{c} p\left(\lambda ,e^{-{\lambda \tau }_{1}},\cdots e^{-{\lambda \tau }_{m}}\right)=\lambda^{n}+{p_{1}}^{\left(0\right)}\lambda^{n-1}+\cdots {p_{n-1}}^{\left(0\right)}\lambda +{p_{n}}^{\left(0\right)}\\ +\left[{p_{1}}^{\left(1\right)}\lambda^{n-1}+\cdots {p_{n-1}}^{\left(1\right)}\lambda +{p_{n}}^{\left(1\right)}\right]e^{-{\lambda \tau }_{1}}+\cdots \\ +\left[{p_{1}}^{\left(m\right)}\lambda^{n-1}+\cdots {p_{n-1}}^{\left(m\right)}\lambda +{p_{n}}^{\left(m\right)}\right]e^{-{\lambda \tau }_{m}}, \end{array} \end{array}$$

where *τ*_*i*_ ≥ 0(*i* = 1, ⋯, *m*) and *p*_*j*_^(*i*)^(*j* = 1, ⋯, *n*; *i* = 1, ⋯, *m*) are constants. As (*τ*_1_, *τ*_2_, ⋯*τ*_*m*_) varies, the sum of orders of the zeros of *p*(*λ*, *e*^−*λτ*_1_^, ⋯*e*^−*λτ*_*m*_^) in the open right half plane can change only if a zero appears on or crosses the imaginary axis.

For *T* = 0, Eq. (7) becomes

(24) $$\begin{array}{c} \lambda^{4}+d_{3}\lambda^{3}+d_{2}\lambda^{2}+d_{1}\lambda +d_{0}=0, \end{array}$$

with

(25) $$\begin{array}{c} d_{0}=1/\tau^{4}-a_{22}/\tau^{3}-\left(a_{12}a_{21}+a_{34}a_{43}\right)/\tau^{2}-a_{22}a_{34}a_{43}/\tau +a_{12}a_{21}a_{34}a_{43},\\ d_{1}=4/\tau^{3}-3a_{22}/\tau^{2}-2\left(a_{12}a_{21}+a_{34}a_{43}\right)/\tau +a_{22}a_{34}a_{43},\\ d_{2}=4/\tau^{2}-3a_{22}/\tau -\left(a_{12}a_{21}+a_{34}a_{43}\right),\\ d_{3}=4/\tau -a_{22}. \end{array}$$

By the Routh–Hurwitz criterion, we know all roots of Eq. (24) have negative real parts if the following condition holds

(26) $$\begin{array}{c} \left(\text{H}3\right)d_{i}>0\left(i=0,1,\cdots 3\right),\\ d_{3}d_{2}>d_{1},\\ d_{3}d_{2}d_{1}>d_{1}^{2}+d_{3}^{2}d_{0}. \end{array}$$

Through the analysis above, we have if the conditions (H1)-(H3) hold, system (3) undergoes a Hopf bifurcation at the equilibrium *u*_0_ when transmission delay *T* = *T*_0_.

### 2.2. Direction and Stability of the Hopf Bifurcation

In Section 2.1, we have studied the condition for Hopf bifurcation occurring. However, how the system advances towards the parkinsonian state by Hopf bifurcation is not clear. In order to further study the relationship between Hopf bifurcation and the pathological beta oscillation, we turn to the center manifold theorem and normal form method to judge the direction of Hopf bifurcation and the stability of bifurcation periodic solutions at a critical value *T*_0_. At first, let *v*(*t*) = *u*(*Tt*), *T* = *T*_0_ + *μ*, *μ* ∈ *R*, then the system (4) can be transformed into the following form

(27) $$\begin{array}{c} \dot{v}\left(t\right)=L_{\mu }\left(v_{t}\right)+F\left(\mu ,v_{t}\right), \end{array}$$

where *L*_*μ*_ : *C*⟶*R*^4^, *F* : *R* × *C*⟶*R*^4^are given by

(28) $$\begin{array}{c} L_{\mu }\left(v_{t}\right)=\left(T_{0}+\mu \right)\left[B_{1}\left(v_{t}\left(0\right)\right)+B_{2}\left(v_{t}\left(-1\right)\right)\right],\\ F\left(\mu ,v_{t}\right)=\left(T_{0}+\mu \right)f\left(v_{t}\left(-1\right)\right)=\left(T_{0}+\mu \right)\left(f_{2}\left(v_{t}\left(-1\right)\right)+f_{3}\left(v_{t}\left(-1\right)\right)\right), \end{array}$$

with

(29) $$\begin{array}{c} f_{2}\left(v_{t}\left(-1\right)\right)={\left(f_{2S},f_{2G},f_{2E},f_{2I}\right)}^{T}=\left(\begin{array}{c} c_{S}\left(w_{GS}^{2}v_{2}^{2}\left(t-1\right)+w_{CS}^{2}v_{3}^{2}\left(t-1\right)-2w_{GS}w_{CS}v_{2}\left(t-1\right)v_{3}\left(t-1\right)\right)\\ c_{G}\left(w_{SG}^{2}v_{1}^{2}\left(t-1\right)+w_{GG}^{2}v_{2}^{2}\left(t-1\right)-2w_{SG}w_{GG}v_{1}\left(t-1\right)v_{2}\left(t-1\right)\right)\\ c_{E}w_{CC}^{2}v_{4}^{2}\left(t-1\right)\\ c_{I}w_{CC}^{2}v_{3}^{2}\left(t-1\right) \end{array}\right), \end{array}$$

(30) $$\begin{array}{c} f_{3}\left(v_{t}\left(-1\right)\right)={\left(f_{3S},f_{3G},f_{3E},f_{3I}\right)}^{T}=\left(\begin{array}{c} e_{S}\left(w_{CS}^{3}v_{3}^{3}\left(t-1\right)-w_{GS}^{3}v_{2}^{3}\left(t-1\right)+3w_{CS}^{2}w_{GS}v_{3}^{2}\left(t-1\right)v_{2}\left(t-1\right)-3w_{GS}^{2}w_{CS}v_{2}^{2}\left(t-1\right)v_{3}\left(t-1\right)\right)\\ e_{G}\left(w_{SG}^{3}v_{1}^{3}\left(t-1\right)-w_{GG}^{3}v_{2}^{3}\left(t-1\right)+3w_{SG}^{2}w_{GG}v_{1}^{2}\left(t-1\right)v_{2}\left(t-1\right)-3w_{GG}^{2}w_{SG}v_{2}^{2}\left(t-1\right)v_{1}\left(t-1\right)\right)\\ -e_{E}w_{CC}^{3}v_{4}^{3}\left(t-1\right)\\ e_{I}w_{CC}^{3}v_{3}^{3}\left(t-1\right) \end{array}\right), \end{array}$$

(31) $$\begin{array}{c} c_{X}=\frac{-8X^{*}\left(M_{X}-X^{*}\right)\left(2X^{*}-M_{X}\right)}{\tau M_{X}^{4}}, \end{array}$$

(32) $$\begin{array}{c} e_{X}=\frac{32X^{*}\left(M_{X}-X^{*}\right)\left(M_{X}^{2}+6X^{*2}-6M_{X}X^{*}\right)}{3\tau M_{X}^{6}}\;\left(X=S,G,E,I\right). \end{array}$$

By the Riesz representation theorem, there exists a function *η*(*θ*, *μ*), *θ* ∈ [−1, 0], such that

(33) $$\begin{array}{c} L_{\mu }\left(\varphi \right)=\int_{-1}^{0}d\eta \left(\theta ,\mu \right)\varphi \left(\theta \right),\varphi \in C. \end{array}$$

In fact, we can choose *η*(*θ*, *μ*) = (*T*_0_ + *μ*)[*B*_1_*δ*(*θ*) + *B*_2_*δ*(*θ* + 1)], where *δ*(*θ*) is the Dirac delta function, i.e.,

(34) $$\begin{array}{c} \delta \left(\theta \right)=\left\{\begin{array}{c} 1,\;\theta =0\\ 0,\;\theta \neq 0 \end{array}\right.. \end{array}$$

For *φ* ∈ *C*^1^([−1, 0], *R*^4^), , we define

(35) $$\begin{array}{c} A\left(\mu \right)\varphi =\left\{\begin{array}{c} \frac{d\varphi }{d\theta },-1\leq \theta <0\\ \int_{-1}^{0}d\eta \left(\theta ,\mu \right)\varphi \left(\theta \right),\theta =0 \end{array}\right., \end{array}$$

(36) $$\begin{array}{c} R\left(\mu \right)\varphi =\left\{\begin{array}{c} 0,-1\leq \theta <0\\ F\left(\mu ,\varphi \right),\theta =0 \end{array}\right.. \end{array}$$

Then, the system (27) can be transformed into the following operator equation:

(37) $$\begin{array}{c} \dot{v}\left(t\right)=A\left(\mu \right)v_{t}+R\left(\mu \right)v_{t}. \end{array}$$

The adjoint operator *A*^∗^ of *A* is given by

(38) $$\begin{array}{c} A^{*}\left(\mu \right)\psi =\left\{\begin{array}{c} -\frac{d\psi }{ds},0\leq s<1\\ \int_{-1}^{0}d\eta^{T}\left(s,\mu \right)\psi \left(-s\right),s=0 \end{array}\right.. \end{array}$$

According to the discussion in Section 2.1, we know that ±*iω*_0_*T*_0_ are eigenvalues of *A*(0) and *A*^∗^(0), let *q*(*θ*) = (1, *χ*, *β*, *γ*)^*T*^*e*^*iT*_0_*ω*_0_*θ*^(−1 < *θ* ≤ 0) be the eigenvectors of *A*(0) corresponding to eigenvalue *iω*_0_*T*_0_, and*q*^∗^(*s*) = (1/*ρ*)(1, *χ*^∗^, *β*^∗^, *γ*^∗^)^*T*^*e*^*iT*_0_*ω*_0_*s*^(0 ≤ *s* < 1) be the eigenvectors of *A*^∗^(0) corresponding to the eigenvalue −*iω*_0_*T*_0_. With a simple computation, we can obtain

(39) $$\begin{array}{c} \chi =\frac{a_{21}e^{-i\omega_{0}T_{0}}}{i\omega_{0}+1/\tau -a_{22}e^{-i\omega_{0}T_{0}}},\\ \beta =\frac{1/\tau +i\omega_{0}}{a_{13}e^{-i\omega_{0}T_{0}}}-\frac{a_{12}a_{21}e^{-i\omega_{0}T_{0}}}{a_{13}\left(1/\tau +i\omega_{0}-a_{22}e^{-i\omega_{0}T_{0}}\right)},\\ \gamma =\frac{a_{43}}{a_{13}}-\frac{a_{12}a_{21}a_{43}e^{-2i\omega_{0}T_{0}}}{a_{13}\left(1/\tau +i\omega_{0}\right)\left(1/\tau +i\omega_{0}-a_{22}e^{-i\omega_{0}T_{0}}\right)},\\ \chi^{*}=\frac{-i\omega_{0}+1/\tau }{a_{21}e^{i\omega_{0}T_{0}}},\\ \beta^{*}=\frac{\left(-i\omega_{0}+1/\tau \right)a_{13}e^{i\omega_{0}T_{0}}}{{\left(-i\omega_{0}+1/\tau \right)}^{2}-a_{34}a_{43}e^{2i\omega_{0}T_{0}}},\\ \gamma^{*}=\frac{a_{34}a_{13}e^{2i\omega_{0}T_{0}}}{{\left(-i\omega_{0}+1/\tau \right)}^{2}-a_{34}a_{43}e^{2i\omega_{0}T_{0}}}. \end{array}$$

And from the definition of the bilinear inner product

(40) $$\begin{array}{c} \langle \psi \left(s\right),\varphi \left(\theta \right)\rangle =\bar{\psi }\left(0\right)\varphi \left(0\right)-\int_{\theta =-1}^{0}\int_{\xi =0}^{\theta }\bar{\psi }\left(\xi -\theta \right)d\eta \left(\theta ,0\right)\varphi \left(\xi \right)d\xi , \end{array}$$

we have

(41) $$\begin{array}{c} \bar{\rho }=\left(1+\chi {\bar{\chi }}^{*}+\beta {\bar{\beta }}^{*}+\gamma {\bar{\gamma }}^{*}\right)+T_{0}e^{-i\omega_{0}T_{0}}\left(a_{21}{\bar{\chi }}^{*}+\left(a_{12}+a_{22}{\bar{\chi }}^{*}\right)\chi +\left(a_{13}+a_{43}{\bar{\gamma }}^{*}\right)\beta +a_{34}{\bar{\beta }}^{*}\gamma \right), \end{array}$$

such that 〈*q*^∗^, *q*〉 = 1, $\langle q^{*},\bar{q}\rangle =0$.

In the following, we use the center manifold theorem to simplify the system. Note that at the Hopf bifurcation point, the corresponding linear system has a pair of pure imaginary eigenvalues *λ* = ±*iω*_0_, and all other eigenvalues have strictly negative real parts. So the whole infinite-dimensional state space *C* could be decomposed into two complementary subspaces, namely, *C* = *E*^*C*^ + *E*^*S*^ [30, 31]. Here, *E*^*C*^ is the two-dimensional subspace spanned by the eigenvectors corresponding to ±*iω*_0_, termed the center eigenspace. And *E*^*S*^ corresponds to the subspace complementary to *E*^*C*^, in which the real part of all eigenvalues is negative. Then, based on the center manifold theorem, there exist a two-dimensional center manifold *C*_0__,_ and the dynamical flow of the system (24) on it can be transformed into

(42) $$\begin{array}{c} v_{t}\left(\theta \right)=q\left(\theta \right)z\left(t\right)+\bar{q}\left(\theta \right)\bar{z}\left(t\right)+W\left(z\left(t\right),\bar{z}\left(t\right)\right), \end{array}$$

with

(43) zt=q∗θ,vt,Wzt,z¯t=vtθ−ztqθ−z¯tq¯θ=vtθ−2Reztqθ,

where *z* is the local coordinate for *C*_0_ in the direction of *q* for the solution of Eq.(35), satisfying

(44) z˙t=q∗θ,v˙t=q∗θ,Aμvt+Rμvt=iT0ω0z+q¯∗0F0,Wt,0+2Reztq0,

and $W\left(z\left(t\right),\bar{z}\left(t\right)\right)$ is the nonlinear map from *E*^*C*^ to *E*^*S*^ with

(45) $$\begin{array}{c} W\left(z\left(t\right),\bar{z}\left(t\right)\right)=W_{20}\left(\theta \right)\frac{z^{2}}{2}+W_{11}\left(\theta \right)z\bar{z}+W_{02}\left(\theta \right)\frac{{\bar{z}}^{2}}{2}+\cdots . \end{array}$$

Thus, our system in the (*z*, *W*) plane reads

(46) $$\begin{array}{c} \left\{\begin{array}{c} \dot{z}\left(t\right)={iT}_{0}\omega_{0}z+g\left(z,\bar{z}\right)\\ \dot{W}\left(t\right)=AW+H\left(z,\bar{z}\right) \end{array}\right., \end{array}$$

with

(47) gz,z¯=q∗θ,Rμvt=q¯∗0F0,Wt,0+2Reztq0,Hz,z¯=F0,qz+q¯z¯+W−q∗,F0,qz+q¯z¯+Wq−q¯∗,F0,qz+q¯z¯+Wq¯.

Then, we apply the normal form theory to deduce the Poincare normal form for the Hopf bifurcation, i.e.,

(48) $$\begin{array}{c} \dot{\xi }=i\omega_{0}\xi +c_{1}\left(0\right)\xi {\left|\xi \right|}^{2}+o\left({\left|\xi \right|}^{3}\right), \end{array}$$

where *o*(|*ξ*|^3^) represents all terms of fourth and higher order in |*ξ*|, and

(49) $$\begin{array}{c} c_{1}\left(0\right)=\frac{i}{2\omega_{0}}\left[g_{20}g_{11}-2{\left|g_{11}\right|}^{2}-\frac{{\left|g_{02}\right|}^{2}}{3}\right]+\frac{g_{21}}{2}, \end{array}$$

where *g*_*ij*_(*i* + *j* = 2) and *g*_21_ can be explicitly determined. Let us rewrite $g\left(z,\bar{z},W\right)$ as follows:

(50) $$\begin{array}{c} g\left(z,\bar{z},W\right)=g_{20}\frac{z^{2}}{2}+g_{11}z\bar{z}+g_{02}\frac{{\bar{z}}^{2}}{2}+g_{21}\frac{z^{2}\bar{z}}{2}+o\left({\left|z\right|}^{3}\right), \end{array}$$

with the term *o*(|*z*|^3^) including all terms of fourth and higher order in |*z*|; then, the calculation of *g*_*ij*_(*i* + *j* = 2) is straightforward.

By keeping *F*(0, *v*_*t*_) = *f*_2_(*v*_*t*_(−1)) and inserting $v_{t}\left(\theta \right)=q\left(\theta \right)z\left(t\right)+\bar{q}\left(\theta \right)\bar{z}\left(t\right)$ in Eq. (29), we get

(51) $$\begin{array}{c} g_{20}=\frac{\partial^{2}g\left(0,0\right)}{\partial z^{2}}=\frac{T_{0}}{\bar{\rho }}\left(\frac{\partial^{2}f_{2S}}{\partial z^{2}}+{\bar{\chi }}^{*}\frac{\partial^{2}f_{2G}}{\partial z^{2}}+{\bar{\beta }}^{*}\frac{\partial^{2}f_{2E}}{\partial z^{2}}+{\bar{\gamma }}^{*}\frac{\partial^{2}f_{2I}}{\partial z^{2}}\right),\\ g_{02}=\frac{\partial^{2}g\left(0,0\right)}{\partial {\bar{z}}^{2}}=\frac{T_{0}}{\bar{\rho }}\left(\frac{\partial^{2}f_{2S}}{\partial {\bar{z}}^{2}}+{\bar{\chi }}^{*}\frac{\partial^{2}f_{2G}}{\partial {\bar{z}}^{2}}+{\bar{\beta }}^{*}\frac{\partial^{2}f_{2E}}{\partial {\bar{z}}^{2}}+{\bar{\gamma }}^{*}\frac{\partial^{2}f_{2I}}{\partial {\bar{z}}^{2}}\right),\\ g_{11}=\frac{\partial^{2}g\left(0,0\right)}{\partial z\partial \bar{z}}=\frac{T_{0}}{\bar{\rho }}\left(\frac{\partial^{2}f_{2S}}{\partial z\partial \bar{z}}+{\bar{\chi }}^{*}\frac{\partial^{2}f_{2G}}{\partial z\partial \bar{z}}+{\bar{\beta }}^{*}\frac{\partial^{2}f_{2E}}{\partial z\partial \bar{z}}+{\bar{\gamma }}^{*}\frac{\partial^{2}f_{2I}}{\partial z\partial \bar{z}}\right), \end{array}$$

where

(52) $$\begin{array}{c} \frac{\partial^{2}f_{2S}}{\partial z^{2}}=2c_{S}e^{-2{iT}_{0}\omega_{0}}\left(\chi^{2}w_{GS}^{2}+\beta^{2}w_{CS}^{2}-2\chi \beta w_{GS}w_{CS}\right),\\ \frac{\partial^{2}f_{2G}}{\partial z^{2}}=2c_{G}e^{-2{iT}_{0}\omega_{0}}\left(w_{SG}^{2}+\chi^{2}w_{GG}^{2}-2\chi w_{SG}w_{GG}\right),\\ \frac{\partial^{2}f_{2E}}{\partial z^{2}}=2c_{E}w_{CC}^{2}\gamma^{2}e^{-2{iT}_{0}\omega_{0}},\\ \frac{\partial^{2}f_{2I}}{\partial z^{2}}=2c_{I}w_{CC}^{2}\beta^{2}e^{-2{iT}_{0}\omega_{0}},\\ \frac{\partial^{2}f_{2X}}{\partial {\bar{z}}^{2}}=conj\left(\frac{\partial^{2}f_{2X}}{\partial z^{2}}\right)\;\left(X=S,G,E,I\right),\\ \frac{\partial^{2}f_{2S}}{\partial z\partial \bar{z}}=c_{S}\left[2\chi \bar{\chi }w_{GS}^{2}+2\beta \bar{\beta }w_{CS}^{2}-\left(\chi \bar{\beta }+\bar{\chi }\beta \right)w_{GS}w_{CS}\right],\\ \frac{\partial^{2}f_{2G}}{\partial z\partial \bar{z}}=c_{G}\left[2w_{SG}^{2}+2\chi \bar{\chi }w_{GG}^{2}-\left(\chi +\bar{\chi }\right)w_{GS}w_{CS}\right],\\ \frac{\partial^{2}f_{2E}}{\partial z\partial \bar{z}}=2c_{E}w_{CC}^{2}\gamma \bar{\gamma },\\ \frac{\partial^{2}f_{2I}}{\partial z\partial \bar{z}}=2c_{I}w_{CC}^{2}\beta \bar{\beta }. \end{array}$$

On the other hand, considering

(53) $$\begin{array}{c} g_{21}=\frac{\partial^{3}g\left(0,0\right)}{\partial z^{2}\partial \bar{z}}=\frac{\partial^{3}}{\partial z^{2}\partial \bar{z}}\langle q^{*},f_{3}\rangle +\frac{\partial^{3}}{\partial z^{2}\partial \bar{z}}\langle q^{*},f_{2}\rangle , \end{array}$$

one can calculate *g*_21_ by two parts. The calculation of $\left(\partial^{3}/\partial z^{2}\partial \bar{z}\right)\langle q^{*},f_{3}\rangle$ is as straightforward as the calculation of *g*_20_. Insertion of $v_{t}\left(\theta \right)=q\left(\theta \right)z\left(t\right)+\bar{q}\left(\theta \right)\bar{z}\left(t\right)$ into Eq. (30) yields

(54) $$\begin{array}{c} \frac{\partial^{3}}{\partial z^{2}\partial \bar{z}}\langle q^{*},f_{3}\rangle =\frac{T_{0}}{\bar{\rho }}\left(\frac{\partial^{3}\left(f_{3S}\right)}{\partial z^{2}\partial \bar{z}}+{\bar{\chi }}^{*}\frac{\partial^{3}\left(f_{3G}\right)}{\partial z^{2}\partial \bar{z}}+{\bar{\beta }}^{*}\frac{\partial^{3}\left(f_{3E}\right)}{\partial z^{2}\partial \bar{z}}+{\bar{\gamma }}^{*}\frac{\partial^{3}\left(f_{3I}\right)}{\partial z^{2}\partial \bar{z}}\right), \end{array}$$

where

(55) $$\begin{array}{c} \frac{\partial^{2}f_{3S}}{\partial z^{2}\partial \bar{z}}=6e_{S}e^{-{iT}_{0}\omega_{0}}\left(\beta^{2}\bar{\beta }w_{CS}^{3}-\chi^{2}\bar{\chi }w_{GS}^{3}+w_{CS}^{2}w_{GS}\left(2\beta \bar{\beta }\chi +\beta^{2}\bar{\chi }\right)-w_{GS}^{2}w_{CS}\left(2\chi \bar{\chi }\beta +\chi^{2}\bar{\beta }\right)\right),\\ \frac{\partial^{2}f_{3G}}{\partial z^{2}\partial \bar{z}}=6e_{G}e^{-{iT}_{0}\omega_{0}}\left(w_{SG}^{3}-\chi^{2}\bar{\chi }w_{GG}^{3}+w_{SG}^{2}w_{GG}\left(2\chi +\bar{\chi }\right)-w_{GG}^{2}w_{SG}\left(2\chi \bar{\chi }+\chi^{2}\right)\right),\\ \frac{\partial^{2}f_{3E}}{\partial z^{2}\partial \bar{z}}=-6e_{E}e^{-{iT}_{0}\omega_{0}}\gamma^{2}\bar{\gamma }w_{CC}^{3},\\ \frac{\partial^{2}f_{3I}}{\partial z^{2}\partial \bar{z}}=6e_{I}e^{-{iT}_{0}\omega_{0}}\beta^{2}\bar{\beta }w_{CC}^{3}. \end{array}$$

The calculation of $\left(\partial^{3}/\partial z^{2}\partial \bar{z}\right)\langle q^{*},f_{2}\rangle$ involves the map $W\left(z,\bar{z}\right)$ from the subspace *E*^*C*^ to its complementary subspace *E*^*S*^. With the second equation of Eq. (46) in mind, Taylor expansion of $H\left(z,\bar{z}\right)$ is as follows:

(56) $$\begin{array}{c} H\left(z,\bar{z}\right)=H_{20}\frac{z^{2}}{2}+H_{11}z\bar{z}+H_{02}\frac{{\bar{z}}^{2}}{2}+o\left({\left|z\right|}^{2}\right), \end{array}$$

and then by comparing the corresponding coefficients of Eq. (56) and Eq. (45), we have

(57) $$\begin{array}{c} \left(A-2{iT}_{0}\omega_{0}\right)W_{20}=-H_{20},\\ {AW}_{11}=-H_{11},\\ \left(A+2{iT}_{0}\omega_{0}\right)W_{02}=-H_{02}, \end{array}$$

where *W*_20_(−1) and *W*_11_(−1) can be computed based on the following equations, respectively.

$W_{20}\left(\theta \right)=\left({ig}_{20}/T_{0}\omega_{0}\right)q\left(0\right)e^{{iT}_{0}\omega_{0}\theta }+\left(i{\bar{g}}_{20}/3T_{0}\omega_{0}\right)\bar{q}\left(0\right)e^{-{iT}_{0}\omega_{0}\theta }+E_{1}e^{2{iT}_{0}\omega_{0}\theta },$

(58) $$\begin{array}{c} W_{11}\left(\theta \right)=-\frac{{ig}_{11}}{T_{0}\omega_{0}}q\left(0\right)e^{{iT}_{0}\omega_{0}\theta }+\frac{i{\bar{g}}_{11}}{T_{0}\omega_{0}}\bar{q}\left(0\right)e^{-{iT}_{0}\omega_{0}\theta }+E_{2}, \end{array}$$

with

(59) E1=2e−2iT0ω02iω0+1/τ−a12e−2iω0T0−a13e−2iω0T00−a21e−2iω0T02iω0+1/τ−a22e−2iω0T000002iω0+1/τ−a34e−2iω0T000−a43e−2iω0T02iω0+1/τ−1cSwGS2χ2+wCS2β2−2wGSwCSχβcGwSG2+wGG2χ2−2wSGwGGχcEwCC2γ2cIwCC2β2,E2=−1/τa12a130a21−1/τ+a220000−1/τa3400a43−1/τ−1cSwGS2χ2+wCS2β2−2wGSwCSχβ¯+χ¯βcGwSG2+wGG2χ2−4wSGwGGReχcEwCC2γ2cIwCC2β2.

Then, inserting

(60) $$\begin{array}{c} v_{t}\left(\theta \right)=q\left(\theta \right)z\left(t\right)+\bar{q}\left(\theta \right)\bar{z}\left(t\right)+\frac{W_{20}\left(\theta \right)}{2}z{\left(t\right)}^{2}+W_{11}\left(\theta \right)z\left(t\right)\bar{z}\left(t\right)+\frac{W_{02}\left(\theta \right)}{2}\bar{z}{\left(t\right)}^{2} \end{array}$$

into Eq. (29) to get

(61) $$\begin{array}{c} \frac{\partial^{3}}{\partial z^{2}\partial \bar{z}}\langle q^{*},f_{2}\rangle =\frac{T_{0}}{\bar{\rho }}\left(\frac{\partial^{3}\left(f_{2S}\right)}{\partial z^{2}\partial \bar{z}}+{\bar{\chi }}^{*}\frac{\partial^{3}\left(f_{2G}\right)}{\partial z^{2}\partial \bar{z}}+{\bar{\beta }}^{*}\frac{\partial^{3}\left(f_{2E}\right)}{\partial z^{2}\partial \bar{z}}+{\bar{\gamma }}^{*}\frac{\partial^{3}\left(f_{2I}\right)}{\partial z^{2}\partial \bar{z}}\right), \end{array}$$

where

(62) $$\begin{array}{c} \frac{\partial^{2}f_{2S}}{\partial z^{2}\partial \bar{z}}=2c_{S}\left(\begin{array}{c} w_{GS}^{2}\left(2\chi e^{-{iT}_{0}\omega_{0}}W_{11}^{\left(2\right)}\left(-1\right)+\bar{\chi }e^{{iT}_{0}\omega_{0}}W_{20}^{\left(2\right)}\left(-1\right)\right)\\ +w_{CS}^{2}\left(2\beta e^{-{iT}_{0}\omega_{0}}W_{11}^{\left(3\right)}\left(-1\right)+\bar{\beta }e^{{iT}_{0}\omega_{0}}W_{20}^{\left(3\right)}\left(-1\right)\right)\\ -2w_{GS}w_{CS}\left(\chi e^{-{iT}_{0}\omega_{0}}W_{11}^{\left(3\right)}\left(-1\right)+\beta e^{-{iT}_{0}\omega_{0}}W_{11}^{\left(2\right)}\left(-1\right)\right)\\ -w_{GS}w_{CS}\left(\bar{\chi }e^{{iT}_{0}\omega_{0}}W_{20}^{\left(3\right)}\left(-1\right)+\bar{\beta }e^{{iT}_{0}\omega_{0}}W_{20}^{\left(2\right)}\left(-1\right)\right) \end{array}\right),\\ \frac{\partial^{2}f_{2\text{G}}}{\partial z^{2}\partial \bar{z}}=2c_{G}\left(\begin{array}{c} w_{SG}^{2}\left(2e^{-{iT}_{0}\omega_{0}}W_{11}^{\left(1\right)}\left(-1\right)+e^{{iT}_{0}\omega_{0}}W_{20}^{\left(1\right)}\left(-1\right)\right)\\ +w_{GG}^{2}\left(2\chi e^{-{iT}_{0}\omega_{0}}W_{11}^{\left(2\right)}\left(-1\right)+\bar{\chi }e^{{iT}_{0}\omega_{0}}W_{20}^{\left(2\right)}\left(-1\right)\right)\\ -2w_{SG}w_{GG}\left(e^{-{iT}_{0}\omega_{0}}W_{11}^{\left(2\right)}\left(-1\right)+\chi e^{-{iT}_{0}\omega_{0}}W_{11}^{\left(1\right)}\left(-1\right)\right)\\ -w_{SG}w_{GG}\left(e^{{iT}_{0}\omega_{0}}W_{20}^{\left(2\right)}\left(-1\right)+\bar{\chi }e^{{iT}_{0}\omega_{0}}W_{20}^{\left(1\right)}\left(-1\right)\right) \end{array}\right),\\ \frac{\partial^{2}f_{2\text{E}}}{\partial z^{2}\partial \bar{z}}=2c_{E}w_{CC}^{2}\left(2\gamma e^{-{iT}_{0}\omega_{0}}W_{11}^{\left(4\right)}\left(-1\right)+\bar{\gamma }e^{{iT}_{0}\omega_{0}}W_{20}^{\left(4\right)}\left(-1\right)\right),\\ \frac{\partial^{2}f_{2\text{I}}}{\partial z^{2}\partial \bar{z}}=2c_{I}w_{CC}^{2}\left(2\beta e^{-{iT}_{0}\omega_{0}}W_{11}^{\left(3\right)}\left(-1\right)+\bar{\beta }e^{{iT}_{0}\omega_{0}}W_{20}^{\left(3\right)}\left(-1\right)\right). \end{array}$$

With *g*_*ij*_(*i* + *j* = 2) and *g*_21_ all available, one can obtain *c*_1_(0) as Eq. (49), and the model (3) could be transformed into the Poincare form. Based on the results in Ref [30], the coefficient *μ*(*ε*) which determine the exists of the periodic solution, the period *T*(*ε*) and the nontrivial Floquet exponent near 0 of the periodic solution are given by

(63) $$\begin{array}{c} \mu \left(\varepsilon \right)=\mu_{2}\varepsilon^{2}+O\left(\varepsilon^{3}\right),\\ T\left(\varepsilon \right)=\frac{2\pi }{\omega_{0}}\left(1+T_{2}\varepsilon^{2}\right)+O\left(\varepsilon^{3}\right),\\ \beta \left(\varepsilon \right)=\beta_{2}\varepsilon^{2}+O\left(\varepsilon^{3}\right), \end{array}$$

with

(64) μ2=−Rec10Reλ′,T2=−Imc10+μ2Imλ′ω0,β2=2Rec10.

Therefore, we have the following results:

1. The sign of *μ*_2_ determines the direction of the Hopf bifurcation: if *μ*_2_ > 0 (*μ*_2_ < 0), the Hopf bifurcation is supercritical (subcritical)
2. The sign of *T*_2_ determines the period of the bifurcating periodic solutions: if *T*_2_ > 0 (*T*_2_ < 0), the period increases (decreases)
3. The sign of *β*_2_ determines the stability of the bifurcating periodic solutions: if *β*_2_ < 0 (*β*_2_ > 0), the bifurcation periodic solutions are stable (unstable)

## 3. Results

**Figure 2:**
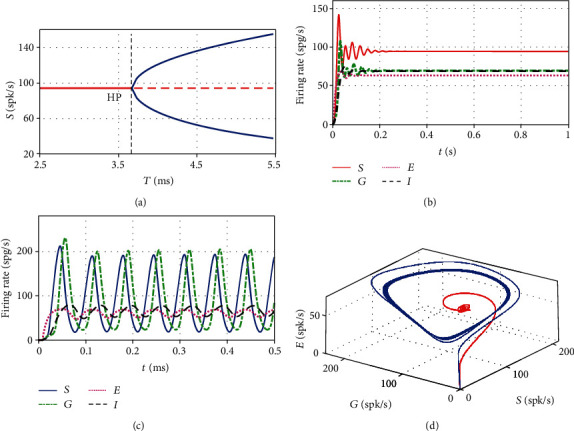
(a) Bifurcation diagram of the firing rate of the STN population *S*(*t*) against the transmission delay *T*. The critical point is consistent with the theoretical result, as indicated by the black dashed line, where the steady state is stable at left and unstable at right. (b) Time series of the firing rate for *T* = 2.5 ms. (c) Time series of firing rate for *T* = 8.0 ms. (d) The corresponding phase diagram in which the red curve corresponds to Figure 2(b), and the blue curve corresponds to Figure 2(c).

**Figure 3:**
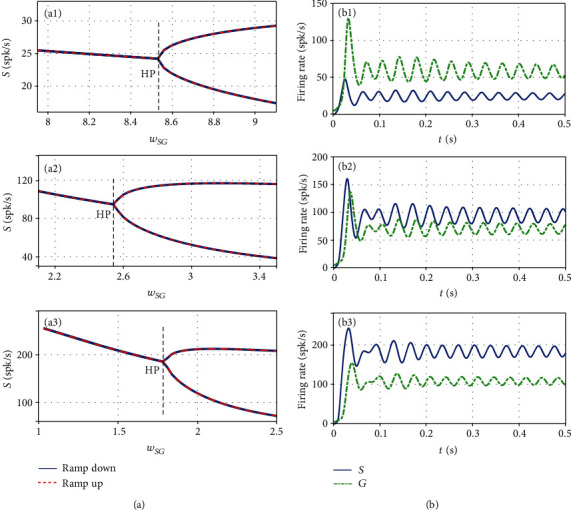
(a) Bifurcation diagrams of the firing rate of the STN population *S*(*t*) against the connection weight *w*_*SG*_ and (b) time series of *S* and *G* at critical points for three selected values of *w*_*CS*_ ((a1)*w*_*CS*_ = 4.0, (a2)*w*_*CS*_ = 6.6, (a3)*w*_*CS*_ = 10.0). The critical points are consistent with the theoretical results, as indicated by the black dashed lines, where the steady states are stable at left and unstable at right.

**Figure 4:**
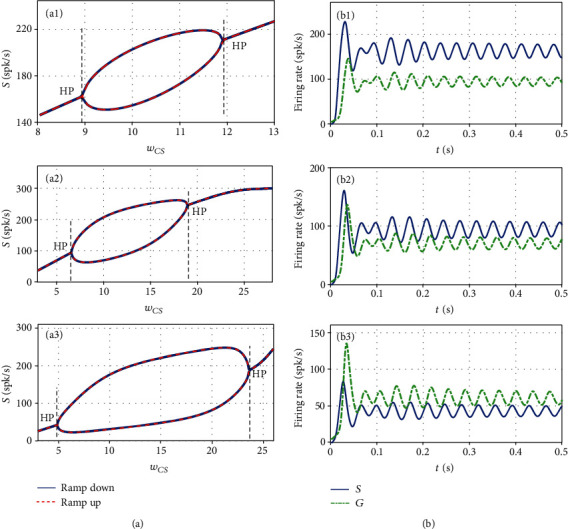
(a) Bifurcation diagrams of the firing rate of the STN population *S*(*t*) against the connection weight *w*_*CS*_and (b) time series of *S* and *G* at the left critical points for three selected values of *w*_*SG*_. ((a1)*w*_*SG*_ = 1.85, (a2)*w*_*SG*_ = 2.56, (a3)*w*_*SG*_ = 5.00). The critical points are consistent with the theoretical results, as indicated by the black dashed lines.

Synaptopathy is the earliest step in the Parkinson's disease cascade [32]. To clarify the effects of synaptic transmission delay and connection strength on the onset of beta oscillations, numerical simulations are implemented with model (3) to validate the theoretical results in the previous deduction, with comparison of analytical predictions of Hopf bifurcation types with the numerically calculated bifurcation diagrams that are presented in Figures 2 - 4. For convenience, we take the parameters from Ref. [24], that is, *w*_*SG*_ = 2.56, *w*_*GS*_ = 3.22, *w*_*CC*_ = 2.75, *w*_*CS*_ = 6.60, *w*_*GG*_ = 0.90, *C* = 277.94 and Str = 40.51. In the calculations, we distinguish between the supercritical and subcritical Hopf bifurcation by ramping up and ramping down the parameter across the critical point with the Euler integration, respectively [33].

### 3.1. The Dependence of Oscillations on Synaptic Transmission Delay

Experimental data suggested that synaptic transmission delay exists between different neuronal populations [34]. And the time delay could often be a source of oscillation. Therefore, we firstly explore the dependence of oscillation onset on the transmission delay in this resonance model. According to the analytic deduction in Section 2, we know that Hopf bifurcation occurs at *T*_0_ = 3.6807ms, with *μ*_2_ = 5.5252 × 10^−4^, *β*_2_ = −1.4874 × 10^−5^, and *T*_2_ = 8.6223 × 10^−6^. Here, the signs of *μ*_2_, *β*_2_, and *T*_2_ imply that the Hopf bifurcation is supercritical, the periodic solution is stable, and the period increases with increasing time delay, respectively. To confirm the theoretical results, let us resort to Figure 2. From the bifurcation diagram (fixed points or local minimums and maximums for each parameter point) against transmission delay in Figure 2(a), we see that the model (3) undergoes a supercritical Hopf bifurcation at *T*_0_ = 3.6807ms, starting from a stable equilibrium point (Figure 2(b)) and entering into a stable limit loop (Figure 2(c)). The two kinds of attractors are usually referred to as a healthy state and an oscillation state, which falls into the beta band here [16]. Moreover, as the time delay increases, the period gradually becomes large. The simulated results are totally consistent with the theoretical analysis, and this enables us to do cross validation throughout Section 3, although we skip the discussion.

Besides, from Figure 2(c), one clearly sees that beta oscillations occur in the excitatory neuronal population of cortex first, and then the basal ganglia resonates at the same frequency. This explains why the model (3) was called as the “resonance” model [24]. Hence, synaptic transmission delay between neuronal populations in the basal ganglia and cortex needs to be sufficiently long to allow them to “charge” and increase their firing rate, which is just coincident with the results in Refs [12, 13, 17].  Figure 2(d) exhibits the corresponding phase portraits, in which the red curve converges to a point, representing the health state, while the blue curve converges to a limit cycle, corresponding to the Parkinsonian state.

### 3.2. The Dependence of Oscillations on Connection Weights

Although most of the parameters in the resonance model could be fixed based on the experimental data in Ref. [25], the synaptic connection weights *w*_*ij*_ cannot be estimated from experimental studies directly, which are usually estimated by fitting the model to experimental recordings. Moreover, it is well known that the depletion of dopamine in basal ganglia maybe related to the synaptic connection strength [35]. As a result, it should be interesting to explore the effect of the synaptic connection strength on the generation of pathological beta oscillation. For this purpose, let us fix the transmission delay *T* = 3.6708ms and show in Figure 3 the bifurcation diagrams of the firing rate against *w*_*SG*_, which controls the strength of the excitatory input from STN to GPe.

As shown in Figure 3, the model (3) undergoes a supercritical Hopf bifurcation when the connection weight *w*_*SG*_ is changed from the healthy to the Parkinsonian parameter. A beta oscillation of small amplitude appears after the destabilization of the steady state, and the oscillation amplitude increases as the weight *w*_*SG*_ enlarges. Thus, pathological beta oscillation could be significantly attenuated or restrained in the resonance model by blocking the STN-GPe connection, which in agreement with the experimental observation reported in Ref. [25]. In addition, as seen from Figures 3(a1)–(a3), the Hopf bifurcation value becomes smaller with increasing *w*_*CS*_, but the mean firing rates of STN and GPe populations get large as the weight *w*_*CS*_ enlarges, as shown in Figures 3(b1)–(b3). It reveals that the excitatory input from the cortex to the subthalamic nucleus also affects the neuronal activity in basal ganglia.

Next, we examine the impact of the connection weight *w*_*CS*_, which links the STN-GPe circuit and cortical circuit, on the beta oscillation onset. Figures 4(a1)–(a3) exhibit the range of the firing rate of the STN population after the initial transient response as a function of the connection weight *w*_*CS*_. It reveals that there are two bifurcation points appear as *w*_*CS*_ increases linearly, consisting with the theoretical prediction. When the excitatory input from the cortex to STN is too strong or too weak, the firing rates of STN and GPe converge to stable equilibrium points. While the beta oscillations are generated when the connection *w*_*CS*_ lies in an moderate region, the amplitude firstly increases and then declines until zero. That may be the reason why blocking the excitatory input to STN could abolish beta oscillation in STN [25]. At the same time, it further demonstrates that the parameter range for beta oscillations gets bigger when the connection *w*_*SG*_ increases. Figures 4(b1)–(b3)) depict the corresponding time series of firing rates of STN and GPe at the left Hopf bifurcation points, respectively.

### 3.3. Codimension Two Bifurcation Analysis

**Figure 5:**
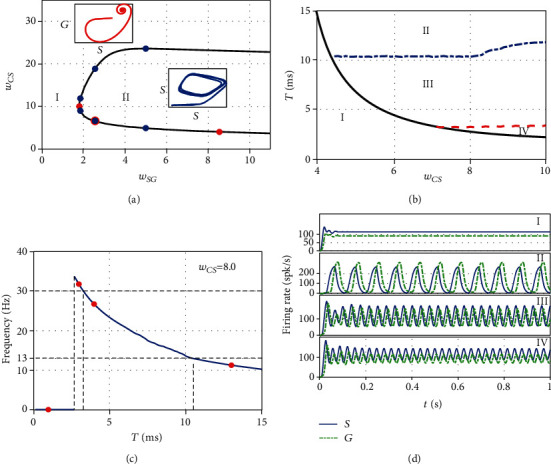
(a) Boundary line of Hopf bifurcation in the parameter space (*w*_*SG*_, *w*_*CS*_), where the red points correspond to Hopf bifurcation points in Figure 3, and the blue points correspond to Hopf bifurcation points in Figure 4. (b) Boundary line of Hopf bifurcation in the parameter space (*w*_*CS*_, *T*), where the black curve is the Hopf bifurcation curve, and the equilibrium point is stable in domain I, corresponding to the healthy-type behavior. The unstable region includes three parts, oscillations with an *α*‐band onset frequency in domain II, a *β*‐band frequency in domain III, and a *γ*‐band frequency in domain IV. (c) The evolution of the oscillation frequency via the synaptic delay *T* with *w*_*CS*_ = 8.0. (d) Time series of *S* and *G* with parameters *w*_*CS*_ = 8.0 and *T* = 1.0ms in the first panel, *T* = 13.0ms in the second panel, *T* = 4.0ms in the third panel, and *T* = 3.0ms in the last panel, which corresponds to the red points in Figure 5(c), respectively.

The above numerical results show that the critical points for Hopf bifurcation in the resonance model can be influenced not only by STN-GPe connection but also the excitatory input from the cortex to STN. To illustrate how the beta oscillation onset depends on both the STN-GPe and cortex-STN connection strength, we depict the codimension-two bifurcation diagram in Figure 5(a). It is clear that when the two-dimensional bifurcation parameter (*w*_*SG*_, *w*_*CS*_) is located in domain I, the system converges to a stable equilibrium point, but excessive oscillations at beta frequencies occur when the parameter is within domain II. Hence, the system always stays at a healthy state for small *w*_*CS*_ or *w*_*SG*_. This is equally to say that the beta oscillations cannot be generated in the single STN-GPe circuit but can originate from interaction among different neuronal population circuits, which is in agreement with Ref [24]. Therefore, blocking the connection between the STN-GPe circuit and cortical circuit may restrain the appearance of beta oscillation.

The codimension-two bifurcation diagram with the connection weight *w*_*CS*_ and the transmission delay *T* is shown in Figure 5(b), where the parameter region is separated by the Hopf bifurcation curve into parts: one is related with steady firing rate and the other is about the oscillatory firing rate. What is more, the oscillatory region can be divided into three parts: alpha oscillation (marked as II with 8-13 Hz oscillation frequencies), beta oscillation (III, 13-30 Hz), and gamma oscillation (IV, larger than 30 Hz). With *w*_*CS*_ = 8.0 fixed, the variation of the nonzero oscillation frequency with transmission delay in Figure 5(c) exhibits the transition among oscillations at gamma, beta, and alpha band frequency in turn, and the firing rate directly evolves from a steady value into the gamma oscillation, but the beta band frequency occupies a more wide region. For intuitiveness, the firing rates of STN and GPe with different band frequencies are exemplified in Figure 5(d). From Figures 5(b) and 5(c), it is clear that a moderate range of synaptic delay is responsible for the emergence of beta oscillation, which contains the model parameters in Refs. [15, 24].

## 4. Discussion

We have investigated critical conditions for pathological beta oscillation onset in the resonance model based on the center manifold theorem and normal form analysis. It is confirmed that the model undergoes a supercritical Hopf bifurcation as the synaptic transmission delay increases, which governs the transitions from the healthy state to the Parkinsonian state. It is found that a strong excitatory connection from STN to GPe is favorable for the generation of beta oscillations, while excessive excitatory input from cortex to STN would suppress beta oscillations. Particularly, the codimension-two bifurcation diagram suggests that the beta oscillation onset depends on the interaction of the STN-GPe circuit and Cortex-STN synaptic connection. Our investigation has demonstrated that a suitable transmission delay is responsible for the emergence of the beta oscillation. The investigation could be inspiring for clinical physician in treating Parkinsonian patients.

In the near future, this study can be extended to generalized models with more biological conditions such as the feedback connection from the STN-GPe circuit to the cortex. Note that the model of this study considers only four populations, namely, the excitatory population and the inhibitory population of cortex, the subthalamic nucleus and globus pallidus external segment, thus, for more insight in this regard, one may consider the more complicated model [21] which can take striatum and globus pallidus internal segment into account as well. In addition, the effect of the synaptic plasticity and the environmental fluctuations on the onset of beta oscillation should also be worthy to be explored.

## Figures and Tables

**Table 1:** The parameter values used in this paper.

Parameter	Value	Parameter	Value
*τ*_*S*_	12.8 ms	*τ*_*G*_	20 ms
*τ*_*E*_	10—20 ms	*τ*_*I*_	10—20 ms
*B*_*S*_	10 spk/s	*B*_*G*_	20 spk/s
*B*_*E*_	0—20 spk/s	*B*_*I*_	0—20 spk/s
*M*_*S*_	300 spk/s	*M*_*G*_	400 spk/s
*M*_*E*_	50—80 spk/s	*M*_*I*_	20—330 spk/s
Str	40.51 spk/s	*C*	277.94 spk/s

## Data Availability

The data of parameters used to support the findings of this study are included within the article.
